# Sufficient Gas and Solvated Ion Transport Assisted Rapid NH_3_ Detection at Room Temperature Through Bionic Olfactory Fibres

**DOI:** 10.1002/EXP.20250609

**Published:** 2026-06-14

**Authors:** Hongyang Liu, Lingyun Xu, Xiaohan Sun, Zhihao Zhao, Qi Song, Weijie Wang, Zhe Chen, Gongmo Xiang, Yupeng Chen, Fanrong Zhao, Xiangyu Jiang, Lei Jiang

**Affiliations:** ^1^ School of Chemistry Beihang University Beijing P. R. China; ^2^ College of Materials Science and Technology Beijing Forestry University Beijing P. R. China; ^3^ Department of Applied Chemistry China Agricultural University Beijing China; ^4^ International Research Institute For Multidisciplinary Science Beihang University Beijing P. R. China; ^5^ Key Laboratory of Bio‐inspired Smart Interfacial Science and Technology of Ministry of Education, School of Chemistry Beihang University Beijing China; ^6^ Key Laboratory of Bio‐inspired Materials and Interfacial Science Technical Institute of Physics and Chemistry Chinese Academy of Sciences Beijing China

**Keywords:** bioinspired integrated sensing system, bionic olfactory sensing, gas transport pathway, ionic signal transmission, wet NH_3_ detection

## Abstract

The human olfactory sensing system, based on ionic signal transmission, is featured with fastness, high efficiency and low energy consumption. However, bionic gas sensing materials exhibit performance limitations compared to materials based on electronic signal transmission. Herein, bionic olfactory fibres are prepared by electrospinning for rapid gas sensing at the ppb level, which consist of confined ionic liquids (ILs) within nano spacing in a polymer matrix. The fibres showed a high response (69.29%) to 500 ppb NH_3_, ultrafast response (4 s) and a low theoretical limit of detection (45 ppb). The excellent sensing performance is attributed to the sufficient gas transport pathways formed by gas convection within the fibrous pore structures. In addition, the rapid transport of solvated ions, caused by the encapsulation of target molecules around ILs in the confined nano spacing, also plays a role, as confirmed by experimental and simulation results. Moreover, bionic olfactory fibres demonstrate excellent gas cyclic stability, mechanical robustness and humidity resistance, which makes them highly suitable for disease diagnosis and seafood spoilage detection in humid environments. Using AI‐driven data analysis on gas response from shrimp spoilage, 95% test accuracy was attained, enabling precise seafood freshness monitoring. This work provides a novel platform for intelligent gas perception through hardware‐software codesign, showing promising potential to create bioinspired integrated sensing systems combining gas and solvated ion transport mediation with AI for decision‐making analysis.

## Graphical abstract

Bionic olfactory fibres with nanoconfined ionic liquids were developed to mimic human olfactory cilia. Benefiting from sufficient gas transport pathways provided by inter‐fibre interconnected pores and rapid transport of solvated ions in the confined nano spacing, bionic olfactory fibres show excellent sensing performance to NH_3_ and demonstrate the promising potential in disease diagnosis and seafood spoilage detection under the assistance of artificial intelligence.

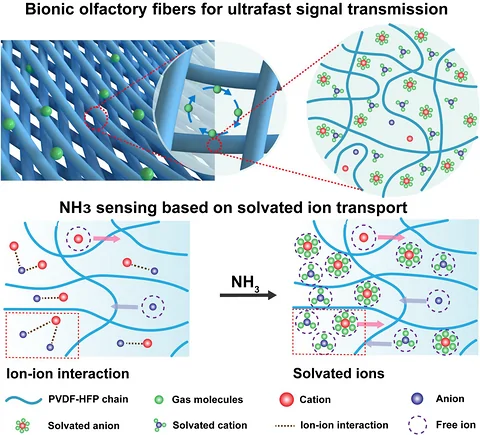

## 1 Introduction

Ammonia (NH_3_) is a common toxic gas that can irritate human respiratory organs (maximum allowable concentration in a workshop is 40 ppm) [1]. Significantly, NH_3_ is a typical diagnostic biomarker for end‐stage renal disease (ESRD), detectable in human exhaled breath at higher concentrations (mean 4.88 ppm) [2]. Furthermore, NH_3_ serves as a crucial indicator of seafood deterioration, stemming from the breakdown of amino acids during spoilage, facilitating food quality monitoring [3]. Currently, sensing materials for NH_3_ based on electronic signal transmission primarily are focused on metal oxides [1, 4], carbon materials [5] and conducting polymers [6, 7, which show shortcomings such as high energy consumption or a poor balance between response/recovery speed and selectivity. Therefore, selective detection of NH_3_ at the ppb level with high sensitivity at room temperature (RT) is a great challenge, especially in complex gas environments or high‐humidity atmospheres [8, 9].

**FIGURE 1:**
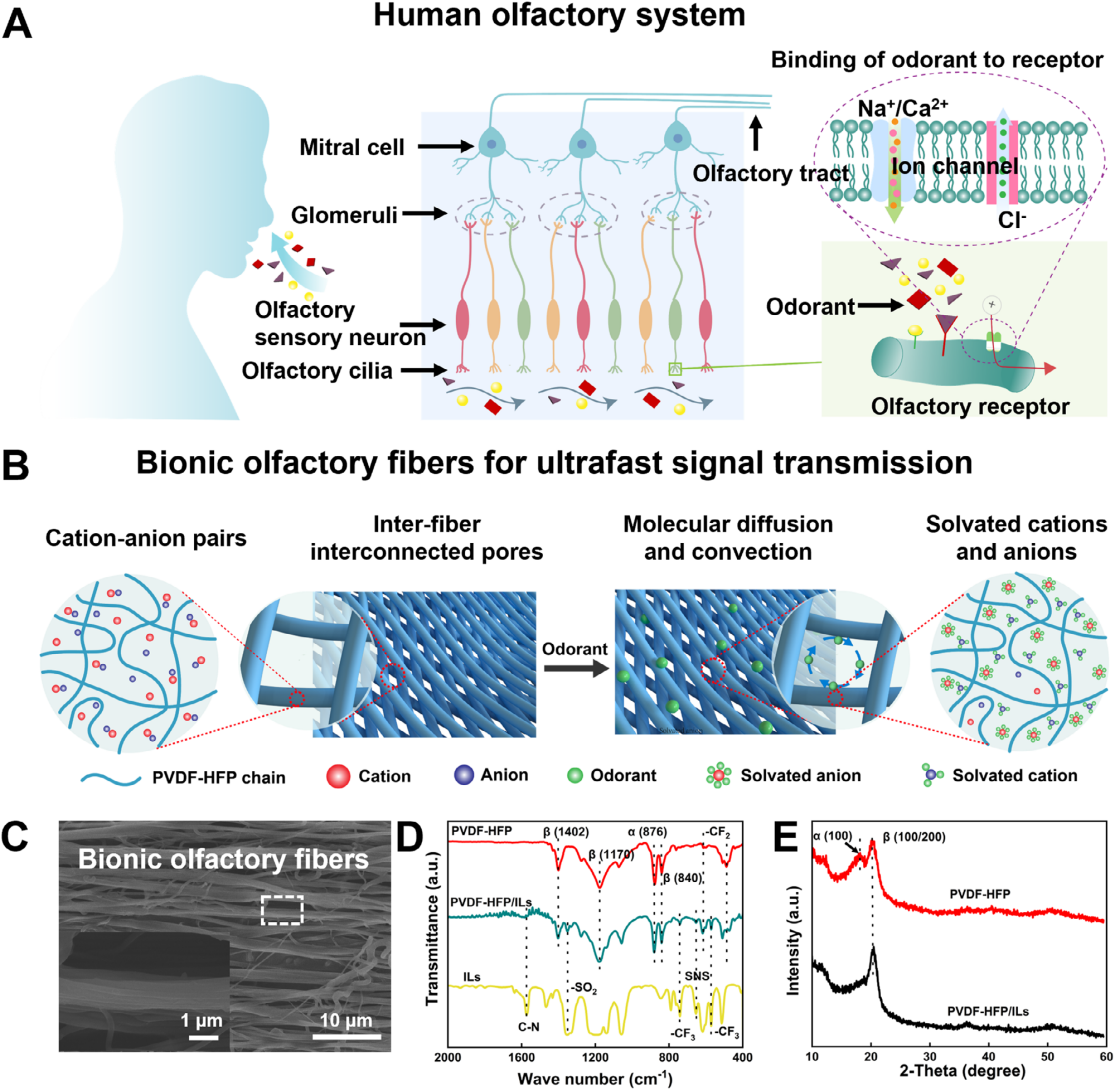
Olfactory fibres inspired by human olfactory cilia. (A) The human olfactory sensing mechanism: The binding of odorants to specific receptors located on olfactory cilia leads to ion transmembrane transport, resulting in the generation of neural signals. (B) Synergistic gas diffusion, convection and confined rapid transport of solvated ions for efficient NH_3_ detection in bionic olfactory fibres. (C) SEM image and the enlarged image of bionic olfactory fibres consisting of PVDF‐HFP and [BMIM][TFSI]. (D) FTIR and E) XRD spectra of the PVDF‐HFP and bionic olfactory fibres.

In nature, numerous organisms have evolved efficient olfactory systems, providing valuable insights for innovative gas sensing methodologies [10, 11, 12]. Humans can distinguish both the aroma of delicious food and the pungent smell of harmful gases using highly sensitive olfactory systems. The specific binding between odorants and olfactory receptors on olfactory cilia induces the transmembrane ion transport, generating neural signals. The signals are transmitted to the glomeruli for a high degree of signal integration in the olfactory bulb and then are sent rapidly and directionally through mitral cells, followed by transmission along the olfactory tract to the cerebral cortex, enabling olfactory sensing (Figure 1A) [13]. Due to rapid response, high sensitivity and low energy consumption of the biological olfactory system, bionic olfactory sensing based on nanofluidic technology has been proposed [14, 15, 16, 17, 18, 19], such as the conically shaped nanochannels grafted with imidazole‐containing polymers [20] and 1‐(4‐amino‐phenyl)‐2,2,2‐trifluoro‐ethanone for CO_2_ sensing [21]. However, the limited diffusion rate from the gas phase to the liquid phase, along with the volatility and fluidity of water‐soluble media, restricts the development of portable bionic olfactory sensing devices. While hydrogels, an emerging material for detecting target gases, have been explored as alternatives, their long‐term water retention capacity is required in various humid environments and the corresponding mass transfer efficiency needs to be further improved [22, 23, 24, 25]. In contrast, ionic liquids (ILs) have emerged as ideal candidates for bionic olfactory sensing due to their nonvolatility, electrochemical stability and structural adjustability [26, 27, 28].

Herein, inspired by human olfactory cilia, we present bionic olfactory fibres with oriented alignment for rapid response toward NH_3_ based on 1‐butyl‐3‐methylimidazolium bis(trifluoromethylsulfonyl)imide ([BMIM][TFSI]) confined within the crystalline domains of poly(vinylidene fluoride‐co‐hexafluoropropylene) (PVDF‐HFP) (Figure 1B). The bionic olfactory fibres showed high and rapid response to NH_3_ at the ppb level, taking advantage of the rapid transport of solvated ions confined in the nano spacing, which is verified by experiment and simulation results. Besides, the porous nanofiber structure enhances the gas mass transfer efficiency, thereby facilitating improved sensing performance. The high sensitivity and selectivity are attributed to the excellent affinity between ILs and NH_3_, which leads to the stronger molecular interaction between the ILs and NH_3_ compared to other gases. Specifically, bionic olfactory fibres showed a high response (69.29%) to 500 ppb NH_3_, ultrafast response (4 s) and a low theoretical limit of detection, denoted as LOD (45 ppb). Moreover, the bionic olfactory fibres exhibited highly reliable NH_3_ detection under 50 gas cycles, 30° bending and 80% relative humidity (RH), respectively, possessing the ability to detect human exhaled ammonia and seafood spoiled ammonia. Furthermore, using artificial intelligence (AI)‐assisted data analysis to analyse gas response data from shrimp spoilage, we achieved 95% accuracy in the test set, enabling precise monitoring of seafood freshness. Eventually, the as‐prepared bionic olfactory fibres with sufficient gas and solvated ion transport and the employment of AI for decision‐making analysis provide a novel avenue to achieve bioinspired integrated sensing systems through hardware‐software codesign, taking a huge step forward in creature‐like intelligent perception.

## 2 Results and Discussion

### 2.1 Construction of Bionic Olfactory Fibres

In the construction of bionic olfactory fibres, [BMIM][TFSI] was selected as the ionic liquid component based on its prominent material merits. Specifically, it exhibits exceptional thermal stability and chemical inertness, which are critical for sustaining consistent sensing baselines and safeguarding the long‐term operational reliability of the fabricated devices. Furthermore, the molecular structure of [BMIM][TFSI] demonstrates high compatibility with the sensing demands for NH_3_, rendering it a suitable candidate for target molecule perception. Bionic olfactory fibres consisting of PVDF‐HFP and [BMIM][TFSI] have been successfully prepared (Figure 1C). The bionic olfactory fibres are smooth and continuous with a uniform diameter of 437 ± 186 nm (Figure S1), showing an oriented network structure which provides rapid ion transport channels and a sufficient gas transport pathway for the binding of target molecules to bionic olfactory fibres [29]. As shown in the Fourier transform infrared (FTIR) spectra of PVDF‐HFP, PVDF‐HFP/ILs (bionic olfactory fibres) and ILs ([BMIM][TFSI]), ‐CF_3_ bending at 568 cm^−1^, S‐N‐S bending at 652 cm^−1^, CH_3_(N) bending at 740 cm^−1^, SO_2_ stretching at 1135 cm^−1^ and CH_3_(N) bending at 1573 cm^−1^ are both observed in the PVDF‐HFP/ILs and ILs [30], indicating that ILs were well combined with the polymer matrix (Figure 1D). Furthermore, the bands at 795 and 976 cm^−1^ assigned to the α‐phase of PVDF‐HFP were suppressed in the PVDF‐HFP/ILs, indicating that the interactions between ions and the PVDF dipoles in the crystalline phase reduce the crystallinity of α‐phase PVDF‐HFP [31]. Additionally, FTIR spectroscopy revealed that upon incorporation of [BMIM][TFSI] into PVDF‐HFP, the CH_3_ vibrational peak at 2967 cm^−^^1^ shifted to 2971 cm^−^^1^, indicating the presence of dipole‐dipole interactions between the electronegative fluorine atoms in the polymer and the cationic imidazolium rings of the IL. Complementarily, the significantly decreased intensity of the observed peaks for the α‐phase PVDF‐HFP (18.16°) further confirmed the interactions (Figure 1E) [32]. The intercalation of ILs into PVDF‐HFP reduces its crystallinity, creating more amorphous channels that facilitate the diffusion of gas molecules toward the active sites of ILs. Also, the existence of N, O and S in the bionic olfactory fibres, which only exist in [BMIM][TFSI], indicates that ILs were dispersed uniformly within the entire polymeric network, as shown by Energy dispersive spectrometer (EDS) (Figure S2). Generally, bionic olfactory fibres with an oriented structure and ILs confined within the nano spacing of the polymer matrix have been successfully prepared for bionic olfactory sensation based on ionic signal transmission [33, 34, 35].

### 2.2 Sensing Performances of Bionic Olfactory Fibres

**FIGURE 2:**
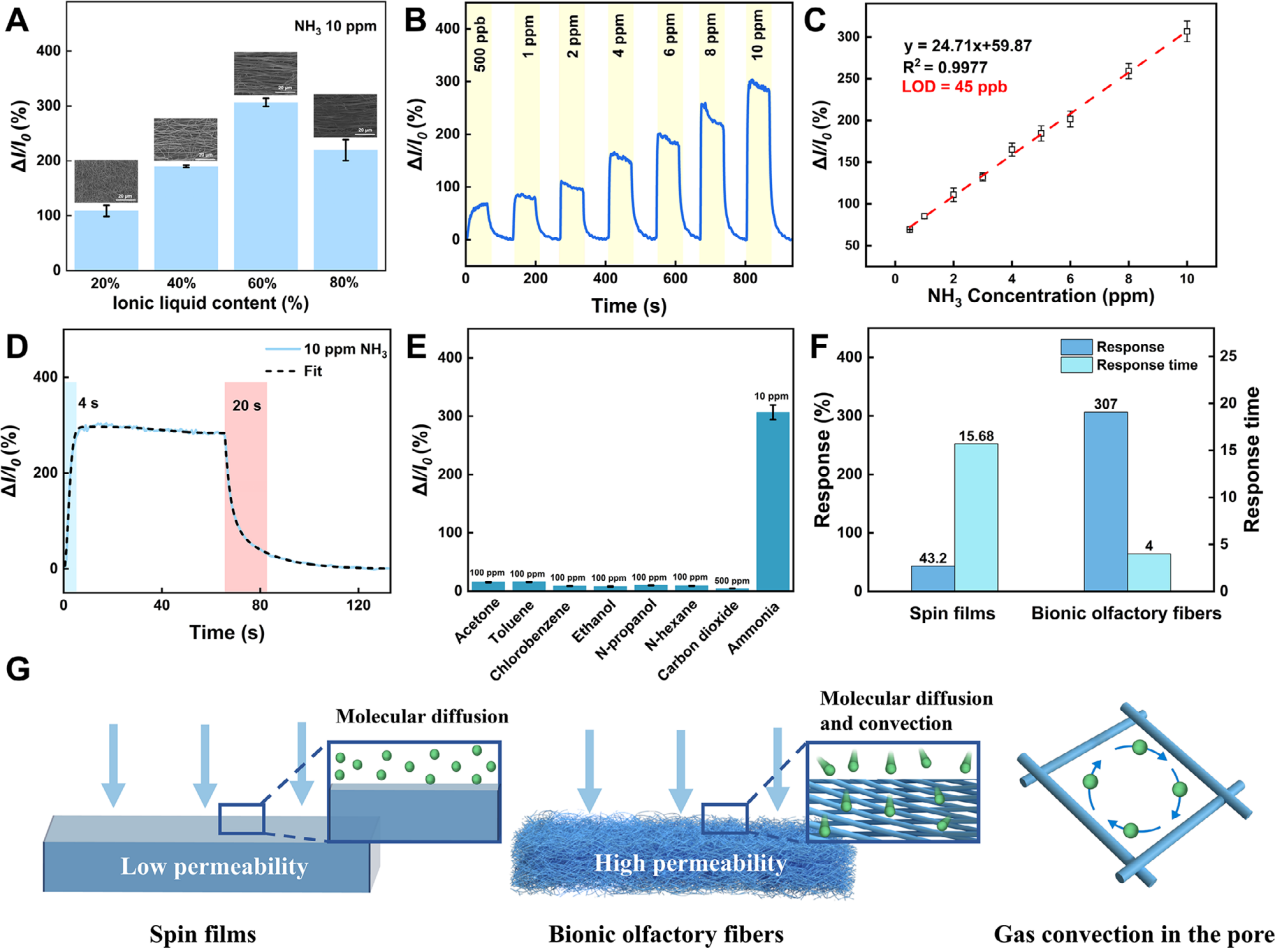
Sensing performance of bionic olfactory fibres. (A) Average response to 10 ppm NH_3_ and SEM images for bionic olfactory fibres with various concentrations of [BMIM][TFSI]. (B) Transient response‐recovery characteristics toward NH_3_ in the range from 0.5 to 10 ppm. (C) Theoretical LOD determined by linear extrapolation from the response slope in the linear region. (D) The response and recovery time to 10 ppm NH_3_. (E) Response to different gases. (F) Response and response time to 10 ppm NH_3_ for spin films and bionic olfactory fibres. (G) Gas mass transfer process for spin films and bionic olfactory fibres.

The impact of IL content on the sensing performance of bionic olfactory fibres was initially investigated. As the IL content increases, the fibre diameter increases. Because of the chargeability and nonvolatility of ILs, aggregation and fusion of fibres can be observed due to the backbuilding of fibres (Figure S3) [36]. Afterwards, the effect of the IL content on the sensing performance was investigated. The response increased from 109% to 307% as the [BMIM][TFSI] content increased from 20% to 60% due to the increased interaction between ions and NH_3_ molecules. However, when the IL content increased from 60% to 80%, the response decreased from 307% to 220%, which might be caused by the increased viscosity of ILs at higher concentrations (Figures 2A and S4) [37].

The dynamic response of bionic olfactory fibres to NH_3_ at different concentrations at RT is shown in Figure 2B. When bionic olfactory fibres were exposed to NH_3_, an obvious positive current change was observed. As the NH_3_ concentration varied from 500 ppb to 10 ppm, the corresponding response increased from 69.29% to 306.21% (Figure S5). Furthermore, a linear correlation was observed between the response and NH_3_ concentration, showing a fitting quality of *R* ^2^ = 0.9977 (Figure 2C). An ultralow LOD of 45 ppb was determined through linear extrapolation from the response slope in the linear region (Table S1 and Figure S6) [38]. The bionic olfactory fibres exhibited a rapid response (4 s) and recovery (20 s) time to 10 ppm NH_3_ (Figure 2D). The high response to 0.5 ppm NH_3_ (69.29%) and rapid response time (4 s) are primarily attributed to sufficient gas pathways for facilitating the binding of NH_3_ with bionic olfactory fibres and oriented alignment structure for shortening ion transport channels. Pure ILs showed a low and unstable response to 10 ppm NH_3_ (24.6%) and slow response time (42 s) due to less ionic dissociation, which further demonstrated the rapid transport of solvated ions in the nano spacing of the polymer matrix (Figure S7) [28, 39]. Furthermore, the bionic olfactory fibres exhibited an ultra‐high response (306.71%) to 10 ppm NH_3_ compared to other analytes, including 100 ppm volatile organic compounds (VOCs) such as acetone (15.25%), toluene (15.56%), chlorobenzene (8.60%), ethanol (7.81%), n‐propanol (9.96%), n‐hexane (8.89%) and 500 ppm carbon dioxide (4.28%), indicating excellent selectivity towards NH_3_ (Figures 2E and S8). Compared to other sensing materials working at RT, bionic olfactory fibres showed a low LOD and rapid response (Table S2), highlighting their advantages for NH_3_ detection.

Further investigation was conducted into the impact of the porous structure of membranes on gas mass transfer. Compared to films prepared by spin‐coating, the bionic olfactory fibres fabricated through electrospinning exhibited higher response and shorter response time (Figures 2F, S9 and S10). Mass transfer primarily relied on the slow diffusion of molecules to the surface of spin films, which suffered from poor gas permeability and the adsorption rate of gases was influenced by the free volume structure of polymer membranes [40]. The bionic olfactory fibres prepared by electrospinning demonstrate a reduced crystallinity degree and an increased proportion of amorphous regions, accompanied by a significant expansion of microscopic free volume. This structural evolution facilitates gas molecule dissolution and diffusion through the polymeric matrix. The attenuated characteristic peak intensities and discernible rightward shift of the characteristic peaks in XRD patterns substantiate the presence of enhanced free volume characteristics in the bionic olfactory fibres (Figure S11). Furthermore, it is suggested that intensified convective transport within the bionic olfactory fibres occurred, leading to a marked enhancement in gas permeability (Figure 2G). The formation of this porous architecture significantly improves membrane permeation performance. Concomitantly with the augmented permeability, surface convective motion is amplified, thereby facilitating environmental gas transfer to the membrane interface. This mass transfer process can also be rationalised through theoretical formulations of mass transfer kinetics. In spin films governed by the solution‐diffusion mechanism, the mass thansfer flux (J_Spin film_) can be mathematically described through the combined application of Fick's laws and Henry's law [41]:

(1) $$\;J_{\mathrm{Spin}\mathrm{film}}=\frac{D_{\mathrm{Poly}\mathrm{mer}}S\Delta P}{L}\;$$

where *D_Polymer_* denotes the diffusion coefficient of the gas within the polymer matrix, *S* is the solubility coefficient, ΔP is the transmembrane pressure differential and *L* is the membrane thickness. In bionic olfactory fibres, gas transport predominantly occurs via direct diffusion through inter‐fibre interconnected pores, with the mass transfer flux (J_Bionic olfactory fibre_) governed by the following relationship [41]:

(2) $$\;J_{\mathrm{Bion}\mathrm{icol}\mathrm{fact}\mathrm{oryf}\mathrm{ibre}}=\frac{D_{0}\varepsilon \Delta P}{\tau L}\;$$

where *D_0_* represents the diffusion coefficient in the free volume domains, *ε* is the porosity (volumetric void fraction), ΔP is the transmembrane pressure differential, *L* is the membrane thickness and *τ* is the tortuosity factor. The gas diffusion coefficient in free volume domains (*D_0_*) significantly exceeds that within polymeric matrices (*D*_Polymer_). This disparity is strategically leveraged by the porous architecture, which enhances gas transport kinetics through the membrane. Consequently, the resultant amplification in mass transfer efficiency directly translates to an accelerated sensing rate toward NH_3_ detection. In summary, orientally arranged fibres with confined ILs based on sufficient gas transport pathways showed a low LOD (45 ppb) and rapid response (4 s) to NH_3_ at RT, enabling the detection of NH_3_ in daily life.

### 2.3 Olfactory Sensing Mechanism

**FIGURE 3:**
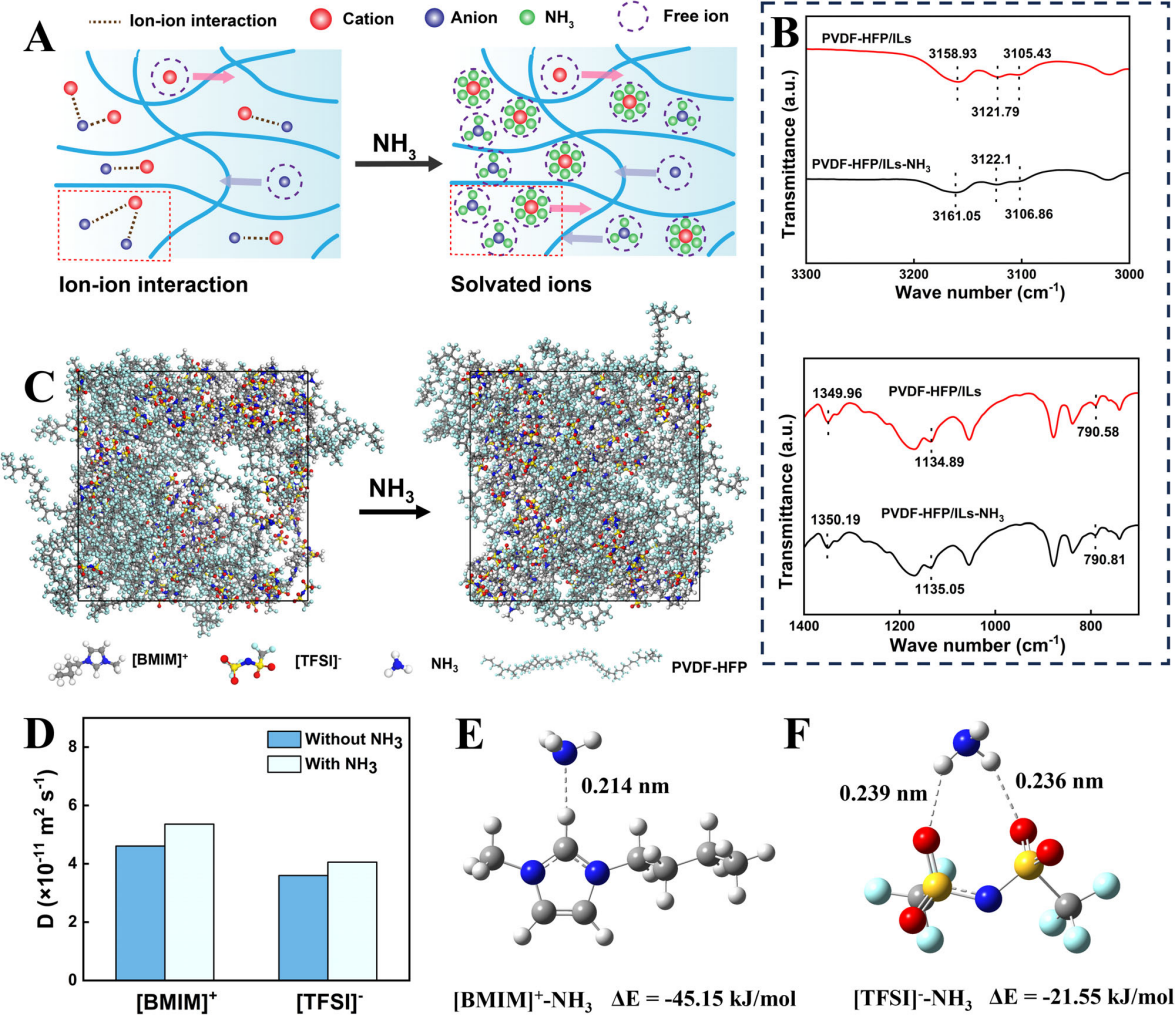
Olfactory sensing mechanism. (A) Schematic illustration of the NH_3_ sensing mechanism of bionic olfactory fibres. (B) Shifts in the FTIR spectra of bionic olfactory fibres when exposed to NH_3_. (C) The snapshots of the motion of [BMIM][TFSI] confined within nano spacing when exposed to NH_3_ in the MD simulation model. (D) Diffusion coefficient (*D*) of [BMIM]^+^ and [TFSI]^−^ without and with NH_3_ treatment, which was obtained from the slopes of mean square displacement (MSD) versus time. (E, F) Optimised structures of [BMIM]^+^‐NH_3_ and [TFSI]^−^‐NH_3_ obtained by ab initio calculations at the B3LYP/6‐311+G* level.

The sensing mechanism of bionic olfactory fibres can be explained by the interaction between the confined ILs and NH_3_ (Figure 3A). ILs exhibit high viscosity due to ion‐ion interactions, which hinder ion diffusion and impair conductivity [42]. Under NH_3_ treatment, the gas molecules dissolve into ILs, which is attributed to the hydrogen bond and van der Waals interactions between NH_3_ and the cations and anions of ILs, resulting in the formation of freely mobile solvated ions. The C2H vibration peak of [BMIM]^+^ shifted from 3105.43 cm^−1^ to 3106.86 cm^−1^, while the C(4,5)H symmetric and asymmetric vibration peaks of [BMIM]^+^ shifted from 3158.93 cm^−1^ and 3121.73 cm^−1^ to 3161.05 cm^−1^ and 3122.1 cm^−1,^ indicating the existence of a strong hydrogen bond interaction between N of NH_3_ and acidic hydrogen of [BMIM]^+^ (Figure 3B) [43, 44, 45]. Simultaneously, the peaks of SO_2_ asymmetric stretching mode and C‐SO_2_‐N bonding mode of [TFSI]^−^ shifted from 1349.96 cm^−1^, 1134.89 cm^−1^ to 1350.15 cm^−1^, 1135.05 cm^−1^, indicating the presence of van der Waals interactions between N of NH_3_ and the O on the [TFSI]^−^ (Figure 3B). At the same time, the weaker peak shift of [TFSI]^−^ indicates that the NH_3_ molecule has a stronger interaction with [BMIM]^+^. The reduced viscosity of the ILs after dissolving gases can be explained by Seddon's equation [46]:

(3) $$\eta =\eta_{S}\;\exp\left(-\frac{\chi_{CS}}{a}\right)$$

where *η* and *η_s_* represent the viscosity of ILs with dissolved gases and pure ILs at 20°C, respectively. *χ_cs_* is the molar fraction of NH_3_, *α* is a constant for the specific ILs. According to the Stokes–Einstein equation, the reduced viscosity of the ILs leads to increased ion diffusivity and conductivity simultaneously, which can be described as follows [47]:

(4) $$D=\frac{\kappa_{B}T}{6\pi \eta r}\;$$

where *D* is the diffusion coefficient of the charged ions, *κ_B_* is the Boltzmann constant, *Τ* is the temperature and *r* is the effective ionic radius. As gas molecules infiltrate into ion channels to interact with cations and anions, solvated ions are formed and the interactions between cations and anions are weakened, leading to a reduction in viscosity [48, 49, 50, 51]. The decreased viscosity promotes ionic diffusion and augments the freely moving ions, increasing ionic conductivity. As indicated by the electrochemical impedance spectroscopy (EIS) results, upon NH_3_ exposure, *R_b_* decreases from 401.72 kΩ to 351.42 kΩ (Figure S12 and Table S3), directly evidencing enhanced bulk ionic conductivity due to ion‐pair dissociation. Concurrently, a significant reduction in *R*_ct_ was observed, indicating that NH_3_ also facilitates the charge transfer kinetics at the interface. Importantly, EIS characterises equilibrium ionic conductivity under alternating current, whereas current variations reflect dynamic ion mobility modulated by external electric fields and polarisation effects under direct current. Consequently, the discrepancies between impedance changes and current fluctuations are not fully commensurate [52]. These observations align with the proposed sensing mechanism, wherein the formation of solvated ions enhances ion mobility [52]. As the NH_3_ concentration increases, a larger number of NH_3_ molecules penetrate the nano‐confined channels. This enhanced molecular infiltration intensifies the disruption of cation‐anion electrostatic interactions, which in turn promotes the dissociation of more ion pairs. The increased dissociation yields a higher population of free ions, ultimately resulting in a more significant change in current (i.e., response). Furthermore, classical field molecular dynamics (MD) simulations are performed to illustrate the motion of [BMIM][TFSI]. When exposed to NH_3_, the mobility of [BMIM]^+^ and [TFSI]^−^ gradually escalates (Figures 3C and S13). The diffusion coefficient of [BMIM]^+^ is predicted to grow from 4.606 × 10^−11^ m^2^ s^−1^ to 5.357 × 10^−11^ m^2^ s^−1^ (increase by 16.30%) when exposed to NH_3_, meanwhile the diffusion coefficient of [TFSI]^−^ is predicted to grow from 3.596 × 10^−11^ m^2^ s^−1^ to 4.054 × 10^−11^ m^2^ s^−1^ (increase by 12.74%) (Figure 3D), indicating that the solvation of ions leads to increased ion diffusivity and conductivity. The larger increase in diffusion coefficient indicates that cations have a stronger interaction with NH_3_ [53]. The optimised structures of [BMIM]^+^‐NH_3_ and [TFSI]^−^‐NH_3_ obtained by the Gaussian 09 package at the B3LYP/6‐311+G* level also confirm the abovementioned results (Figures 3E and 3F). The larger binding energy (*ΔE* = −45.15 kJ/mol) between [BMIM]^+^ and NH_3_ proves that NH_3_ is more prone to associate with acidic hydrogen of [BMIM]^+^, forming solvated ions. Thus, the rapid solvated ion transport in confined nano spacing enables rapid detection of NH_3_ [54]. In summary, during the sensing process, a constant bias voltage of 2 V is applied between the electrodes, establishing a stable and uniform electric field distribution across the bionic olfactory fibres. At this point, the system reaches an initial dynamic equilibrium state, which is manifested as a stable baseline current signal. Upon exposure to NH_3_, the nitrogen atom on the NH_3_ act as a Lewis base site, forming dipole‐dipole interactions or hydrogen bonds with the cations and anions in the bionic olfactory fibres. This intermolecular interaction competes with the original electrostatic attraction between cations and anions, reducing the interaction between cations and anions. As a result, the cation‐anion pairs dissociate partially and the gas molecules coordinate with the cations and anions to form transient solvated ion complexes, where the gas molecules act as solvating shells. This process enhances ion mobility, as evidenced by the increased ionic conductivity observed in EIS measurements (Figure S12). The formation of solvated ions is thermodynamically reversible under ambient conditions, primarily driven by the volatility of the target gas and the dynamic equilibrium between gas adsorption and desorption. When the gas source is removed, the concentration gradient of gas molecules at the sample surface promotes desorption. The weakened cation‐anion interactions are then re‐established due to the absence of competing gas‐phase interactions, leading to the reaggregation of dissociated ions into their original paired states. This dynamic recovery process enables the ionic conductivity to rapidly return to the baseline level, achieving high reversibility of the sensing signal. Other ILs featuring lower viscosity, higher intrinsic conductivity, or shorter alkyl chains may further modulate gas diffusion and sensing kinetics, which could be explored in future studies.

The response of bionic olfactory fibres to other amine substances was further investigated. A good linear response to triethylamine (TEA) in the range of 1–100 ppm was observed, with a LOD of 189 ppb, response time of 21 s and recovery time of 42 s to 10 ppm TEA (Figure S14 and Figure S15). Compared to the FTIR spectra after exposure to NH_3_, a more pronounced peak shift was observed for the C2H vibration peak of [BMIM]^+^ after exposure to TEA (from 3105.43 cm^−1^ to 3103.74 cm^−1^). On the other hand, the C(4,5)H symmetric and asymmetric vibration peaks of [BMIM]^+^ shifted from 3158.93 cm^−1^, 3121.73 cm^−1^ to 3158.33 cm^−1^, 3121.19 cm^−1^, indicating weakened peak shifts. It is suggested that there is a stronger interaction between the basic N on TEA and C2H of [BMIM]^+^ due to the stronger electron‐donating ability of ethyl, but a weakened hydrogen bond interaction between the basic N on TEA and C(4,5)H caused by the larger steric hindrance of ethyl [45]. Additionally, the peaks of SO_2_ asymmetric stretching mode and C–SO_2_–N bonding mode of [TFSI]^−^ shifted from 1349.96 cm^−1^, 1134.89 cm^−1^ to 1350.02 cm^−1^, 1134.87 cm^−1^, with a weakened shift, indicating a weakened interaction (Figure S16). Binding energies calculated via Gaussian 09 at the B3LYP/6‐311+G* level further confirm that [BMIM]^+^ interacts with TEA with a strength comparable to its interaction with NH_3_, whereas the interaction between [TFSI]^−^ and TEA is significantly weaker than that with NH_3_. These simulation results explicitly validate the underlying reason for the inferior response of bionic olfactory fibres to TEA, demonstrating that the interaction strength between ionic liquids and target gases directly governs the sensing response (Figure S17). Such findings provide a theoretical basis for guiding the development of next‐generation ionic signal transmission‐based sensing devices.

### 2.4 Sensing Stability and Application Demonstration

**FIGURE 4:**
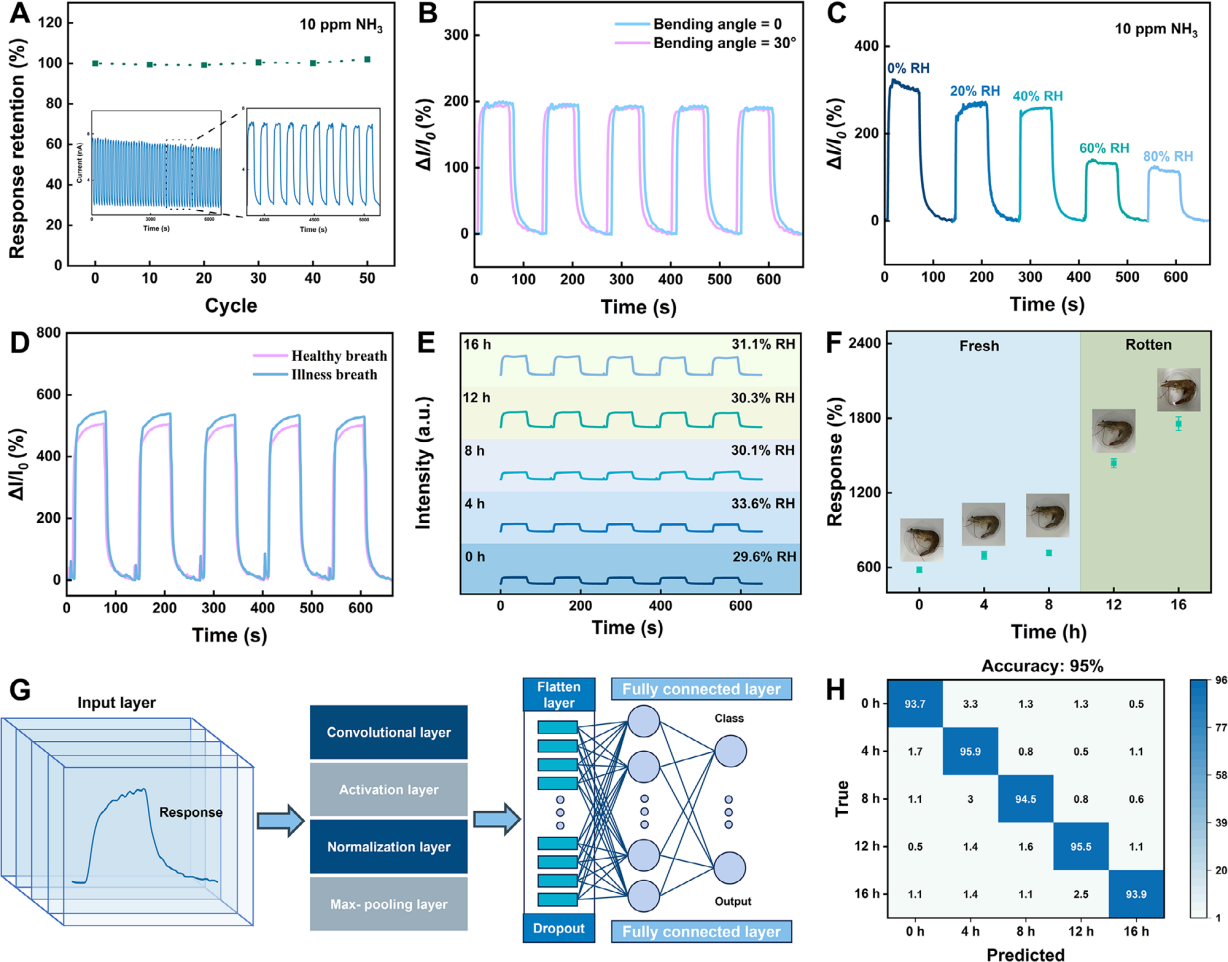
Sensing stability of bionic olfactory fibres. (A) Average response retention during a series of gas cycles to 10 ppm NH_3_. (B) The dynamic response curve of bionic olfactory fibres to 5 ppm NH_3_ with bending angle at 0° and 30°. (C) The dynamic response curve of bionic olfactory fibres to 10 ppm NH_3_ at 0%, 20%,40%, 60% and 80% RH. (D) Response curve of bionic olfactory fibres to healthy exhaled breath and illness breath. (E) The dynamic response curves of bionic olfactory fibres to gases released by shrimps at RT. (F) The response of bionic olfactory fibres increased with the spoilage time of shrimps. (G) The scheme of sensing information processed in the AI‐assisted 1D‐CNN associated learning architecture. (H) Confusion matrix from integrating AI‐assisted 1D‐CNN‐based analysis of shrimp spoilage state.

In order to advance the practical application of the as‐prepared bionic olfactory fibres, the sensing stability was explored, including cyclic stability, mechanical stability and long‐term stability. Slight response changes were observed after 50 cycles of exposure to 10 ppm NH_3_, demonstrating excellent cyclic stability (Figure 4A). Meanwhile, the fibres exhibited no significant response attenuation at 30° bending and maintained stable operation, with only a moderate reduction (≈ 10%) at 45° and a relatively slight decline (≈ 25%) at 60° that did not compromise normal functionality. These findings collectively demonstrate the excellent mechanical stability of the bionic olfactory fibres (Figures 4B and S18). Further tests on the bionic olfactory fibres revealed that the response variation to 10 ppm NH_3_ over 14 days remained within 5%, demonstrating their high reliability (Figure S19). Considering the ubiquitous presence of water vapour in the atmosphere, the impact of RH on the sensing performance of fibres was further investigated (Figure 4C) [55]. The response decreased from 306.71% to 258.51% as the RH increased from 0% to 40%, because the partial solvation of ILs induced by water molecules can weaken the interaction between NH_3_ and ILs. Despite the hydrophobicity of PVDF‐HFP (contact angle of 133.15 ± 0.07°), a portion of water molecules would still enter the polymer matrix and interact with the ILs, as the matrix's hydrophobicity primarily inhibits macroscopic water permeation rather than suppressing interfacial interactions involving microscopic water molecules (Figure S20). Upon interaction with water molecules, distinct shifts of ILs were observed in the FTIR spectra at characteristic vibration peaks. The C2H vibration peak of [BMIM]^+^ shifted from 3105.43 cm^−1^ to 3101.33 cm^−1^, while the C(4,5)H symmetric and asymmetric vibration peaks of [BMIM]^+^ shifted from 3158.93 and 3121.73 cm^−1^ to 3161.20 and 3120.79 cm^−1^ (Figure S21), indicating that the ILs can bind with water molecules via ion‐dipole interactions [56]. Furthermore, the impedance value decreases with the increase of the RH, indicating increased conductivity (Figure S22). It is suggested that partial water molecules permeate into the PVDF‐HFP matrix to surround [BMIM]^+^ and [TFSI]^−^ to weaken ion pair interactions. Under 10 ppm wet NH_3_ with 20% to 80% RH, the impedance value of fibres is further reduced due to enhanced solvation process induced by interactions with NH_3_ (Figure S23). Even at 80% RH, the fibres still exhibit a high response (125.90%) to 10 ppm NH_3_, with a LOD of 123 ppb, demonstrating their suitability for humid environments (Figure S24). Subsequently, specific application scenarios in wet conditions, such as disease monitoring through human exhaled breath analysis (over 90% RH) and seafood spoilage detection (30%–40% RH), were conducted capitalising on their excellent NH_3_ detection performance. NH_3_ serves as a critical biomarker for ESRD, necessitating sensitive detection in high‐humidity settings. Human exhaled gases were collected and subsequently injected with 500 ppb NH_3_ as illness breath. Bionic olfactory fibres can produce significant differences in response between healthy breath and illness breath, enabling disease monitoring through human exhaled breath analysis (Figure 4D). Another application involves detecting NH_3_ in the assessment of seafood freshness. In this study, a batch of fresh shrimps was purchased and stored in a vacuum desiccator at RT. To achieve nondestructive monitoring of the spoilage level, a bag of gases in the desiccator was extracted and tested every 4 h. The dynamic response curves of bionic olfactory fibres to gases released by shrimps are recorded (Figure 4E). With the increase in spoilage time, the response gradually increased. A significant enhancement occurred at 12 h, showing a response of 1438.3%. Meanwhile, visible signs of red discolouration and decay were observed on the shrimp, which corresponded with the sensing results obtained from the periodic sampling (Figure 4F). This is also consistent with the results reported in the literature [57]. This correlation validates the effectiveness of fibres in detecting seafood spoilage. Furthermore, the response data of shrimp spoilage at different time intervals was used as input for convolutional neural networks (CNN) training (10 samples × 5 stages). During the training of the AI‐assisted 1D‐CNN model for shrimp spoilage state classification, the cross‐entropy loss function was employed. The Adam optimiser was utilised to minimise the loss function and update the model parameters, with a learning rate of 0.0005, a batch size of 64 and a total of 200 training epochs. To prevent overfitting and enhance training efficiency, early stopping and learning rate reduction strategies were implemented (Figure 4G). The model demonstrated a classification accuracy exceeding 95% on the test set, indicating its high effectiveness in discerning different states of shrimp spoilage (Figure 4H). Thus, the bionic olfactory fibres exhibit excellent cyclic stability, mechanical stability and resistance to humidity interference, making them feasible for human disease monitoring and seafood spoilage detection.

## 3 Conclusion

In summary, bionic olfactory fibres based on ionic signal transmission with high sensitivity and rapid response performance were developed through electrospinning, consisting of confined ILs in a nano spacing of the polymer matrix. In particular, bionic olfactory fibres exhibited high response to NH_3_ (69.29% to 500 ppb NH_3_) due to sufficient gas transport pathways provided by the nanofiber structure for more binding sites and exhibited rapid response (4 s) due to the high gas mass transfer effciency driven by gas convection within the fibrous pores and rapid solvated ion transport in confined nano spacing. Moreover, bionic olfactory fibres have the possibility of practical application, exhibiting good gas cyclic stability (50 cycles), mechanical stability (30° bending) and resistance to humidity interference (80% RH). Notably, bionic olfactory fibres can detect human exhaled ammonia and seafood spoilage ammonia. When the gas response data from shrimp spoilage are processed using an AI‐assisted 1D‐CNN, it achieves a remarkable 95% accuracy on the test set. This highlights the potential of bionic olfactory fibres in precise monitoring of seafood freshness levels. It is anticipated that the construction of bionic olfactory fibres based on sufficient gas and solvated ion transport and the employment of AI for decision‐making analysis provides a versatile foundation for advancing ionic sensing materials and low‐power neuromorphic devices. With the innovations of functionalised ion engineering and heterostructured confinement strategy, bioinspired integrated sensing systems with precise gas perception and gas‐involved brain‐like computing functions will be developed to address the technical bottlenecks of the gas sensing field, such as limited gas selectivity and response.

## 4 Experimental Section

*Materials*: 1‐butyl‐3‐methylimidazolium bis(trifluoromethylsulfonyl)imide ([BMIM][TFSI], >99%), poly(vinylidene fluoride‐co‐hexafluoropropylene (PVDF‐HFP, *M_w_* ∼ 400,000 g mol^−1^), N,N‐dimethylformamide (DMF, >99.8%) were purchased from Sigma Aldrich. All the reagents were used without further modification unless specially mentioned. Interdigitated electrodes on polyethene glycol terephthalate (PET) substrates with 10 pairs, 0.1 mm distance and 0.1 mm width were purchased from Shengshi Jinding Co., Ltd.

*Construction of bionic olfactory fibres*: The bionic olfactory fibres were fabricated by solution blending and subsequent electrospinning. First, 1 g PVDF‐HFP was dissolved in 3 g DMF, followed by stirring for 12 h at RT and the addition of 0.6 g [BMIM][TFSI] and further stirring for 12 h to obtain the homogeneous PVDF‐HFP/ILs solution. Second, PVDF‐HFP/ILs solution was poured into a 10 mL injection syringe after removing air bubbles and the voltage of 18 kV was applied between the roller collector and spinneret. The PVDF‐HFP/ILs bionic olfactory fibres with oriented alignment were collected at interdigitated electrodes on the roller collector at a speed of 750 rpm. Finally, bionic olfactory fibres were obtained after drying in an oven for 24 h at 80°C [58].

*Characterisation*: The structure of bionic olfactory fibres was observed using a scanning electron microscope (SEM) (S4800, Hitachi, Japan). X‐ray diffraction (XRD) peaks of bionic olfactory fibres (PVDF‐HFP/ILs) and PVDF‐HFP were carried out for investigating the internal structure through an X‐ray diffractometer (D8 focus, Bruker, Germany) using a Cu K_α_ radiation source. Energy dispersive X‐ray spectroscopy (EDS) was used to analyse the elemental distribution of bionic olfactory fibres through a field emission scanning electron microscope (FE‐SEM) (Apreo S LoVac, Thermo Scientific, America). An OCA25 contact angle system (OCA25, Data Physics, Germany) was used to record the surface contact angles of water droplets at ambient temperature. A Fourier transform infrared spectrometer (FTIR) (Nicolet iS20, Thermo Scientific, America) were used to measure the spectra of PVDF‐HFP/ILs, PVDF‐HFP and ILs and explore interactions between PVDF‐HFP/ILs and NH_3_. Electrochemical impedance spectroscopy (EIS) was performed by an electrochemical workstation (CHI‐760E, Shanghai Chenhua Instruments Co., Ltd, China). The frequency of EIS ranged from 10^5^ to 0.1 Hz.

*Gas sensing measurements*: A gas delivery system was designed to control the gas flow for measuring the current changes in bionic olfactory fibres. 100 ppm NH_3_ was collected in a gas bag and then was dynamically diluted by a 310i dynamic dilution gas mixer (Tianjin Huayi Technology Co., Ltd.) with N_2_ to produce target gases of different concentrations. In the initial step, only N_2_ was introduced at a consistent flow rate of 1000 mL/min for 1 min. Subsequently, in the second step, N_2_ and the targeted high‐concentration gas were introduced at varying flow rates while ensuring that the total flow rate remained constant at 1000 mL/min. The RH was adjusted through a humidity control device on the 310i dynamic dilution gas mixer. The signal was accurately measured utilising a semiconductor parameter analyser (4200‐SCS, Keithley, America), employing the I‐t curve mode (with constant voltage output) and applying a voltage of 2 V to the sample electrode. The response was determined using the following equation:

(5) $$\mathrm{Response}\;\;\left(\%\right)=\left(I-I_{0}\right)/I_{0}\;\times 100\%$$

where *I_0_* and *I* are the current before and after exposure to NH_3_, respectively. The response time was defined as the time required to reach 90% of the equilibrium response and the recovery time was defined as the time required to recover 90% of the equilibrium response. For the application demonstration of disease monitoring through human exhaled breath analysis, two bags of exhaled human breath were collected. One of them was added with 0.5 ppm of NH_3_ as the illness breath and the other one was used as a healthy breath sample for testing. For the application demonstration of seafood spoilage detection, a batch of fresh Chinese white shrimps was purchased and stored in a vacuum desiccator at RT. Gas emissions from the shrimps were collected every 4 h as sample gases using a diaphragm pump, while the vacuum dryer was replenished with fresh air. Use a 310i dynamic dilution gas mixer for gas intake and monitor gas humidity.

*Molecular dynamics simulation details*: The density functional theory (DFT) calculations were performed using the Dmol3 module in Material Studio software with the B3LYP functional, custom Grimme DFT‐D parameters and DNP 4.4 basis set. ESP charges were applied to optimise all molecules ([BMIM][TFSI], NH_3_, PVDF‐HFP unit and PVDF‐HFP, which has 5 units) [59, 60]. The *k*‐points were set at Gamma (1 × 1 × 1) and the convergence tolerance was set at 1.0 × 10^−6^ Ha, 2.0 × 10^−3^ Ha/Å and 5.0 × 10^−3^ Å for energy, maximum force and maximum displacement, respectively.

Three electrolyte models were constructed for molecular dynamics (MD) simulations: a box with NH_3_ consisting of 20 5‐unit PVDF‐HFP, 80 [BMIM][TFSI] and 5 NH_3_ molecules; a box without NH_3_ consisting of 20 5‐unit PVDF‐HFP, 80 [BMIM][TFSI]. All simulations were conducted using the Forcite module with the COMPASS force field, with the exception that the cation and anion charges were scaled by 0.7 following similar previous studies [61, 62]. The solution models were equilibrated in the NPT ensemble using the Berendsen barostat with a 0.1 ps decay for 20 ps, 0.0001 Gpa stress and a Nose thermostat at 298 K [63, 64]. After equilibration, production runs were carried out in an NVT ensemble of 200 ps to ensure that the solution system was in equilibrium.

*AI‐assisted 1D‐CNN model design and training procedure*: The AI‐assisted 1D‐CNN neural network architecture comprises several key layers for effective feature extraction and classification. In the first layer, a 1D convolutional layer (conv1) with 256 output channels employs a kernel size of 5, a stride of 1 and padding of 1. This layer extracts local features from the input data, introducing nonlinearity through the rectified linear unit (ReLU) activation function. Subsequently, a batch normalisation layer (bn1) operates on the output of 256 channels, aiding in reducing internal covariate shifts and providing regularisation benefits. Following this, a max pooling layer (max‐pool1) with a pooling kernel size of 2 and a stride of 2 reduces the dimensionality of the data while retaining essential features. The second 1D convolutional layer (conv2) consists of 512 output channels, a kernel size of 3, a stride of 1 and padding of 1, performing additional feature extraction with ReLU activation. Batch normalisation (bn2) is applied to the output of 512 channels, mirroring the structure of the earlier batch normalisation layer. A subsequent max pooling layer (max‐pool2) with a pooling kernel size of 2 and a stride of 2 further reduces dimensionality. The third 1D convolutional layer (conv3) consists of 1024 output channels, a kernel size of 3, a stride of 1 and padding of 1, performing further feature extraction with ReLU activation. Batch normalisation (bn3) is applied to the output of 1024 channels. A subsequent max pooling layer (max‐pool3) with a pooling kernel size of 2 and a stride of 2 further reduces dimensionality. The flattening layer (flatten) transforms the multidimensional data into one dimension for seamless input into the fully connected layers. To prevent overfitting, a dropout layer with a dropout rate of 0.4 randomly omits a fraction of neurons during forward propagation. This is followed by two bidirectional LSTM layers. Then, a dropout layer with a dropout rate of 0.5 randomly omits a fraction of neurons during forward propagation. Then, a dropout layer with a dropout rate of 0.6 randomly omits a fraction of neurons during forward propagation. Then, a dense layer (fc1) with 2048 output features employs the ReLU activation function. Batch normalisation (bn4) is applied to the output of 2048 channels. To prevent overfitting, a dropout layer with a dropout rate of 0.7 randomly omits a fraction of neurons during forward propagation. Then, a dense layer (fc2) with 1024 output features employs the ReLU activation function. Batch normalisation (bn5) is applied to the output of 1024 channels. To prevent overfitting, a dropout layer with a dropout rate of 0.6 randomly omits a fraction of neurons during forward propagation. Finally, the output layer (fc3) uses the softmax activation function to produce probabilities for the five classes of shrimp spoilage states. This comprehensive architecture balances feature extraction, nonlinearity and regularisation to facilitate effective model training and classification.

The cross‐entropy loss function was employed during the training phase of the AI‐assisted 1D‐CNN model. The formula used for calculating cross‐entropy loss is as follows:

(6) $$\mathrm{Cross}-\mathrm{entropy}\;\mathrm{loss}\;=-\sum y_{i}*\log y_{i}^{\prime }$$

where *y_i_* is the true label and *y_i_'* is the predicted probability for the *i*‐th class.

The cross‐entropy loss was minimised through the gradient descent algorithm provided by the Adam optimiser, and L2 regularisation with a regularisation factor of 0.001 was employed to prevent the model from overfitting to the training data and enhance its generalisation capability. The model was trained for 200 epochs with a batch size of 64. Early stopping was implemented to halt training if the validation loss did not improve for 30 epochs, ensuring the model does not overfit. The learning rate was reduced on a plateau, reducing it by a factor of 0.5 if the validation loss did not improve for 20 epochs, with a minimum learning rate of 1*e* ^−7^ (Figure 4G).

## Conflicts of Interest

The authors declare no conflict of interest. Lei Jiang is a member of the *Exploration* editorial board, and he was not involved in the handling or peer review process of this manuscript.

## Supporting information




**Supporting File**: exp270190‐sup‐0001‐SuppMat.docx.

## Data Availability

The data that support the findings of this study are available from the corresponding author upon reasonable request.
