# Supplementary material for: Sufficient Gas and Solvated Ion Transport Assisted Rapid NH3 Detection at Room Temperature Through Bionic Olfactory Fibres
Source: Exploration (Beijing). 2026 Jun 14;6(4):20250609. doi: 10.1002/EXP.20250609 (PMC13394991; doi:10.1002/EXP.20250609)
Supplement: Supplementary file 1 [file EXP2-6-20250609-s001.docx]

Supporting Information

**Sufficient Gas and Solvated Ion Transport Assisted Rapid NH_3_ Detection at Room Temperature through Bionic Olfactory Fibres**

Hongyang Liu, Lingyun Xu, Xiaohan Sun, Zhihao Zhao, Qi Song, Weijie Wang, Zhe Chen, Gongmo Xiang, Yupeng Chen*, Fanrong Zhao*, Xiangyu Jiang*, Lei Jiang

H. Y. Liu, L. Y. Xu, X. H. Sun, Z. H. Zhao, Q. Song, W. J. Wang, Z. Chen, G. M. Xiang

School of Chemistry, Beihang University, Beijing 100191, P. R. China.

E-mail: jiangxy@buaa.edu.cn

Prof. X. Y. Jiang

International Research Institute for Multidisciplinary Science, Beihang University, Beijing 100191, P. R. China.

E-mail: jiangxy@buaa.edu.cn

Prof. Y. P. Chen

College of Materials Science and Technology, Beijing Forestry University, Beijing 100083, P. R. China.

E-mail: ypchen0727@buaa.edu.cn

Prof. F. R. Zhao

Department of Applied Chemistry, China Agricultural University, Beijing 100193, China

E-mail: zhaofanrong@cau.edu.cn

Prof. L. Jiang

Key Laboratory of Bio-inspired Smart Interfacial Science and Technology of Ministry of Education, School of Chemistry, Beihang University, Beijing100191, China.

Key Laboratory of Bio-inspired Materials and Interfacial Science, Technical Institute of Physics and Chemistry, Chinese Academy of Sciences, Beijing 100190, China.

E-mail: jianglei@buaa.edu.cn

**Figure S1.** Particle size analysis of the bionic olfactory fibers with 60% IL.

**Figure S2.** EDS mapping analysis for the bionic olfactory fibers.

**Figure S3.** SEM images of the bionic olfactory fibers with different IL content, including A) 20 wt%, B) 40 wt%, C) 60 wt% and D) 80 wt%.

**Figure S4.** Dynamic response curves to 10 ppm NH_3_ for bionic olfactory fibers with various concentrations of [BMIM][TFSI].

**Figure S5.** Dynamic response curves to NH_3_ from 0.5 ppm to 10 ppm for bionic olfactory fibers.

**Calculation of noise level (RMS_Noise_) and limit of detection (LOD)**

1. Execute linear fit for the response curve versus gas concentration of the sensor and then extract the slope value (sensitivity) and standard error in the linear regime, as shown in Figure S5 (left plots).

2. Take N = 11 data points at the baseline before gas exposure (*Y_i_*).

3. Plot the data (Δ*I/I_0_* (%) versus Time (sec)) and then execute Polynomial fit (5th order) as shown in Figure S5 (right plots).

4. Take statistical parameters and regular residual (*Yi-Ῡ*) of polynomial fit and calculate the root-mean squared deviation (*RMS_Noise_*) and LOD with Equation S1 and S2. The results were shown in Table S1.

${RMS}_{Noise}({ppm}^{-1})=\sqrt{{V_{X}^{2}}/{(N-1)}}$ where $V_{X}^{2}=\sum{(Y_{i}-\bar{Y})}^{2}$ (Equation S1)

$Limit of detection \left( ppm \right)=3\times{{RMS}_{Noise}}/{Slope}$ (Equation S2)

**Figure S6.** Fitting plots of the bionic olfactory fibers. Left plots are linear fitted, right plots are 5th order polynomial fitted.

**Table S1.** Polynomial fitting data of the bionic olfactory fibers.

| **Time (sec)** | **Y_i_** | **Y_i_-**$\bar{\mathbf{Y}}$ | **(Y_i_-**$\bar{\mathbf{Y}}$**)^2^** |
| --- | --- | --- | --- |
| **400** | **15.01041** | **-0.04485** | **0.00201** |
| **410** | **12.3767** | **0.22529** | **0.05076** |
| **420** | **10.78786** | **-0.34689** | **0.12033** |
| **430** | **9.4819** | **0.00881** | **7.76838E-5** |
| **440** | **8.11213** | **0.32472** | **0.10545** |
| **450** | **6.62084** | **0.21811** | **0.04757** |
| **460** | **5.19442** | **-0.85737** | **0.73508** |
| **470** | **3.80073** | **0.48156** | **0.2319** |
| **480** | **2.58318** | **0.17773** | **0.03159** |
| **490** | **1.39056** | **-0.25383** | **0.06443** |

**Figure S7.** Dynamic response curves to 10 ppm NH_3_ for pure [BMIM][TFSI].

**Figure S8.** Dynamic response curves to different gases for bionic olfactory fibers.

**Table S2.** Comparison of the sensing performance to NH_3_ for different sensing materials working at RT.

| Materials | NH_3_  (ppm) | T  (℃) | Response  (%) | τ_res_/τ_rec_  (s) | RH  (%) | LOD  (ppb) | Refs. |
| --- | --- | --- | --- | --- | --- | --- | --- |
| CsPbBr_3_ | 10 | RT | 958 | 12/16 | 0 ~ 52 | 250 | ^[1]^ |
| MAPbBr_3-x_I_x_ | 50 | RT | 750 | 10/20 | - | 50 | ^[2]^ |
| MAPI | 1 | RT | 55 | 110/130 | 20 ~ 80 | 10 | ^[3]^ |
| S-GO | 10 | RT | 73 | 112/33 | 0 ~ 30 | 500 | ^[4]^ |
| PANI/SrGe_4_O_9_ | 0.2 | RT | 16 | 24/269(0.8 ppm) | 0 ~ 90 | 0.25 | ^[5]^ |
| MoSe_2_ | 1 | 30 | 5.5 | 15/135 | 0 ~ 60 | - | ^[6]^ |
| PANI/NiFe_2_O_4_ | 100 | RT | 3080 | 15/21 | - | 250 | ^[7]^ |
| Ti_3_C_2_T_X_ MXene@TiO_2_/MoS_2_ | 100 | RT | 163.3 | 117/88 | 0 ~ 75 | 500 | ^[8]^ |
| WS_2_@PANI | 100 | RT | 216.3 | 25/39 | 0 ~ 70 | 12.6 | ^[9]^ |
| PANI–ZnO | 100 | RT | 16.95 | 18/87 | - | 5000 | ^[10]^ |
| PANI/Nb_2_CT_x_ | 10 | RT | 74.68 | 218/300 | 0 ~ 87.1 | 20 | ^[11]^ |
| Pd-2-PTN-30 | 100 | RT | 2125 | 124 /264 | - | 200 | ^[12]^ |
| Ti_3_C_2_T_x_/V_2_O_5_/Ag | 10 | RT | 1912 | 8/289 | 25-80 | 1000 | ^[13]^ |
| Pd/CDs@TiO_2_ HNSs | 50 | RT | 61 | 16/216 | 20-80 | 300 | ^[14]^ |
| hBN | 180 | RT | 225 | 250/50 | 20-80 | 9000 | ^[15]^ |
| PVDF-HFP/IL | 10 | RT | 306 | 4/20 | 0 ~ 80 | 44 | This work |

**Figure S9.** Dynamic response curves to 10 ppm NH_3_ for spin films.

**Figure S10.** The response and recovery time of spin films to 10 ppm NH_3_.

**Figure S11.** XRD spectra of the spin films and bionic olfactory fibers.

**Figure S12.** Nyquist plots of bionic olfactory fibers without and with 50 ppm NH_3_ treatment.

**Table S3.** Key parameters extracted from equivalent-circuit fits to Nyquist plots under N_2_ and 50 ppm NH_3_ conditions.

|  | Rb (Ω) | Rct (Ω) | C1 | CPE-1-T | CPE-1-P |
| --- | --- | --- | --- | --- | --- |
| N_2_ | 401720 | 3.7133E6 | 7.448E-12 | 4.74E-8 | 0.54466 |
| NH_3_ | 351420 | 3.4896E6 | 6.942E-12 | 6.4073E-8 | 0.50403 |

**Figure S13.** Mean square displacements of A) [BMIM]^+^ and B) [TFSI]^-^ in bionic olfactory fibers without NH_3_ and with NH_3_.

**Figure S14.** Sensing performance of the bionic olfactory fibers. A) Dynamic responses toward various TEA concentrations ranging from 1 to 100 ppm. B) Theoretical detection limit determined by linear extrapolation from the response slope in the linear region. C) Magnified graph to extract the response/recovery time when exposed to 10 ppm TEA.

**Figure S15.** Dynamic response curves to TEA from 1 ppm to 100 ppm for bionic olfactory fibers.

**Figure S16.** Changes in the infrared spectrum of the bionic olfactory fibers when exposed to TEA.

**Figure S17.** Optimized structures of [BMIM]^+^-TEA and [TFSI]^-^-TEA obtained by ab initio calculations at the B3LYP/6-311+G* level.

**Figure S18.** The dynamic response curve of bionic olfactory fibers to 5 ppm NH_3_ with bending angle at 30°, 45° and 60°.

**Figure S19.** The response of bionic olfactory fibers to 10 ppm NH₃ over a 14-day period.

**Figure S20.** Contact angle to water droplets for PVDF-HFP fibers.

**Figure S21.** Changes in the infrared spectrum of the bionic olfactory fibers when exposed to H_2_O.

**Figure S22.** A) Nyquist plots of bionic olfactory fibers at different RH. B) Ionic conductivity of bionic olfactory fibers at different RH.

**Figure S23.** Nyquist plots of bionic olfactory fibers at different RH without and with NH_3_ treatment, including A) 20% RH, B) 40% RH, C) 60% RH and D) 80% RH.

**Figure S24.** Linear correlation between sensing response and NH_3_ concentrations at 80% RH (R^2^ = 0.9969).

**References**

[1] P. D. Hoat; V. K. Vo; S.-H. Bae; H.-J. Lim; D. T. H. Thao; P. T. Hung; N. M. Hung; N. V. Hoang; J.-H. Lee; Y.-W. Heo, *J. Alloys Compd.,* **2023,** *960*, 170731.

[2] W. Jiao; J. He; L. Zhang, *Sensors Actuators B: Chem.,* **2020,** *309*, 127786.

[3] A. Maity; A. K. Raychaudhuri; B. Ghosh, *Sci. Rep.,* **2019,** *9* (1), 7777.

[4] Y. Yu; Z. Liao; F. Meng; Z. Yuan, *Chemosensors,* **2021,** *9* (8), 220.

[5] Y. Zhang; J. Zhang; Y. Jiang; Z. Duan; B. Liu; Q. Zhao; S. Wang; Z. Yuan; H. Tai, *Sensors Actuators B: Chem.,* **2020,** *319*, 128293.

[6] S. Singh; J. Deb; U. Sarkar; S. Sharma, *ACS Applied Nano Materials,* **2020,** *3* (9), 9375-9384.

[7] N. D. Sonwane; S. B. Kondawar, *Synth. Met.,* **2022,** *284*, 117004.

[8] X. Tian; L. Yao; X. Cui; R. Zhao; T. Chen; X. Xiao; Y. Wang, *J. Mater. Chem. A,* **2022,** *10* (10), 5505-5519.

[9] P. Wang; C. Tang; H. Song; L. Zhang; Y. Lu; F. Huang, *ACS Appl. Mater. Interfaces,* **2024,** *16* (11), 14082-14092.

[10] R. Kaur; S. D. Lawaniya; S. Kumar; N. Saini; K. Awasthi, *Appl. Phys. A,* **2023,** *129* (11), 765.

[11] S. Wang; Y. Jiang; B. Liu; Z. Duan; H. Pan; Z. Yuan; G. Xie; J. Wang; Z. Fang; H. Tai, *Sensors Actuators B: Chem.,* **2021,** *343*, 130069.

[12] P. Li; J. Li; F. Liu; Jiannan; J. Shi; K. Yu; H. Xu; F. Su; K. Wang; S. Li; Y. Zhang, *Chem. Eng. J.,* **2025,** *515*, 163425.

[13] J. Liang; T. Wu; H. Chen; Y. Han; A. Fatima; U. Afzal; Y. Zhang; X. Gao; W. Yan, *ACS Applied Nano Materials,* **2025,** *8* (35), 17109-17120.

[14] X. Wang; M. Sun; Q. Lu; J. Zhang; Y. Wang; Y. Dong; Z. Liu; J. Zhang; L. Du; G. Yin, *Sensors Actuators B: Chem.,* **2026,** *446*, 138632.

[15] B. Das; R. Paul; R. Ghosh, *Sensors Actuators B: Chem.,* **2025,** *444*, 138505.
